# Unlocking Neuromorphic Vision: Advancements in IGZO-Based Optoelectronic Memristors with Visible Range Sensitivity

**DOI:** 10.1021/acsaelm.4c00752

**Published:** 2024-07-05

**Authors:** Maria Elias Pereira, Jonas Deuermeier, Rodrigo Martins, Pedro Barquinha, Asal Kiazadeh

**Affiliations:** † i3N/CENIMAT, Department of Materials Science, NOVA School of Science and Technology and CEMOP/UNINOVA, NOVA University Lisbon, Campus de Caparica, 2829-516 Caparica, Portugal

**Keywords:** IGZO optoelectronic synapse, IGZO memristor, hydrogen doping, visible range detection, spiking neural networks, neuromorphic vision sensors

## Abstract

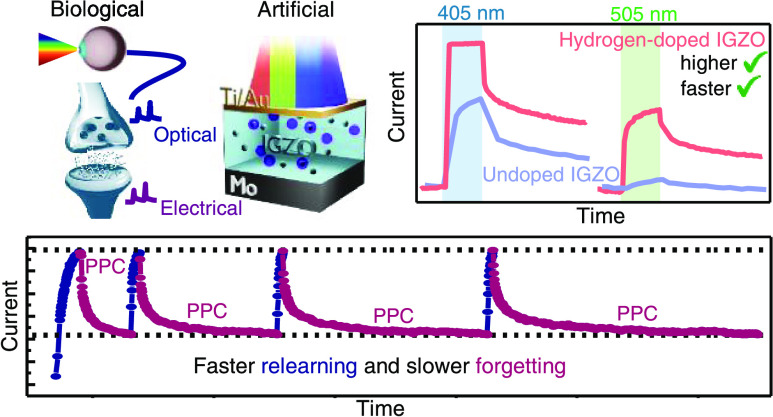

Optoelectronic memristors based on amorphous oxide semiconductors (AOSs) are promising devices for the development of spiking neural network (SNN) hardware in neuromorphic vision sensors. In such devices, the conductance state can be controlled by both optical and electrical stimuli, while the typical persistent photoconductivity (PPC) of AOS materials can be used to emulate synaptic functions. However, due to the large band gap of these materials, sensitivity to visible light (red/green/blue) is difficult to accomplish, which hinders applications requiring color discrimination. In this work, we report a 4 μm^2^ hydrogen-doped (H-doped) indium–gallium-zinc oxide (IGZO) optoelectronic memristor that emulates all of the important rules of SNNs such as short- to long-term memory transition (STM–LTM), paired-pulse facilitation (PPF), spike-time-dependent plasticity (STDP), and learning and forgetting capabilities. By the incorporation of hydrogen gas in the sputtering deposition of IGZO, visible sensitivity was achieved for green and blue wavelengths. Additionally, extremely high light/dark ratios of 179, 93, and 12 are demonstrated for wavelengths of 365, 405, and 505 nm, respectively, due to hydrogen-induced subgap states and device miniaturization. Therefore, the proposed device shows remarkable potential for integration with the pixel circuits of IGZO-based displays with extreme resolution for a true intelligent self-processing display.

## Introduction

In common image-processing systems based on deep-learning, two-dimensional (2D) arrays of photodetectors and image sensors that use semiconductor technology are employed to collect light information as digital inputs. This information is then converted into electric data and saved in a separate memory unit. A processing computing unit with software-based artificial neural networks (ANNs) is required to execute computer vision algorithms, such as object classification.^1,2^ Apart from the obvious delay in the response time due to the necessary data shuffle between the sensors and the processing unit, as well as the high power consumption required to run these complex neural networks, conventional image-processing systems face a significant limitation that further aggravates the previously mentioned challenges. The light sensors capture visual information based on a fixed frame rate, with each recorded frame retaining data from all pixels within the array. On the one hand, a low frame rate may lead to the loss of crucial information; on the other hand, a high frame rate results in the generation of excessive and unnecessary data. In either case, redundant data is shuffled, stored, and processed, coming from the recorded pixels in which no new information was created.^3,4^

An ideal artificial visual system should be able to read, recognize, and perform parallel processing of electrical and optical signals, just as the human brain does. In fact, about 80% of the data collected from the human brain is acquired through light signals by visual perception.^5^ In more detail, our eyes can sense light information and convert it to electrical data, which are subsequently processed and saved in the visual cortex of the brain. Here, the connection between two neurons is referred to as a synapse, involving the transmission of chemical or electrical spikes. The connection between two neurons can become stronger or weaker, which is known as synaptic plasticity, and is closely related to the memory capacity of the human brain. Naturally, short-term memory (STM) denotes a temporary change in the synaptic connection that rapidly decays to its original state once the spike has ended. In contrast, long-term memory (LTM) is the long-lasting, and sometimes permanent, transformation of the synaptic weight.^6,7^

Neuromorphic vision sensors possess the ability to directly detect rapid changes, similar to the human eye. As such, the sensor captures movement as a continuous flow of data rather than a frame-by-frame approach. By allowing each pixel to independently record when triggered, only relevant information is sent to the postprocessing stages.^8−11^ This novel approach not only produces far less data than the conventional one, resulting in increased energy efficiency but also significantly decreases the response time of the system. Emerging optoelectronic memories, using both light and electrical signals as inputs, can behave as sensory artificial synapses with high energy efficiency, low crosstalk, and fast data processing and are, thus, suitable for spiking neural network (SNN) hardware applications in neuromorphic vision sensors.^12,13^

Amorphous oxide semiconductors (AOSs) are an especially interesting class of materials to be employed as photosensitive layers in such devices.^14^ First, AOSs have high flexibility and low-processing temperatures, allowing the use of flexible substrates, which is crucial for internet-of-things (IoT) applications.^15,16^ Additionally, using AOS in optoelectronic memories allows one to benefit from the mature AOS pixel-driver circuit technology employed in commercial flat-panel displays. This holds great promise for a straightforward integration of AOS optoelectronic memories into the neuromorphic system on panel (SoP) technology.

As such, the research community has focused on understanding how to develop an AOS-based synaptic device for neuromorphic vision. The persistent photoconductivity (PPC) and associated relaxation process in oxide semiconductors can be used as a basis to emulate synaptic functions.^17−19^ The precise mechanism for the photogeneration and recombination of charges in oxide semiconductors has not yet been conclusively proven. However, the prevailing theory states that under light stimulation, neutral oxygen vacancies (VOs) are ionized and become positively charged (VO^+^ or VO^2+^). Following light interruption, a slow deionization process takes place, in which electrons slowly move back to VOs.^9,18,20,21^ The activation energy for neutralization of the ionized VOs strongly influences the decay of the photocurrent, following light irradiation. Notably, this activation energy has been observed to be particularly elevated in indium–gallium-zinc oxide (IGZO) at around 0.7 eV.^22^ Moreover, IGZO-based memristors can be seamlessly integrated with the pixel circuits of IGZO-based displays for ultrahigh-resolution SoPs for a new era of neuromorphic display systems. These displays would not only emit light to create images but also possess the ability to sense and process visual information in real time. By detecting the intensity of incident light and discriminating between different colors, dynamically adjusting its resistance, the IGZO optoelectronic memristor emulates synaptic plasticity, enabling the pixel to adapt its response to different lighting conditions and visual stimuli.

Nevertheless, IGZO has a band gap of 3.05 eV, which means that it is mostly sensitive to UV light.^23−25^ The effective tunability of its sensitivity to the visible range is highly important for applications in which color discrimination is necessary. Therefore, strategies such as adding an absorbing layer composed of organic materials,^26^ 2D materials,^27^ quantum dots,^28^ or the engineering of a defective IGZO absorbing layer^29^ have been successful in 3-terminal phototransistors used as synaptic devices. However, this heterostructure approach has its own drawbacks such as high device-to-device (D2D) variability due to increased process complexity, high off-currents (dark current), which then decrease the total photocurrent, and worse overall transistor performance. Hydrogen doping (H-doping) has also been suggested for visible range detection either in a double-layer structure^30^ or by spontaneous doping within one of the interfaces of the device.^31^ The hydrogen atoms incorporated in the IGZO serve as electron donors, which increase the concentration of free electrons^32−34^ and create subgap states in the IGZO layer with different energy levels that can be stimulated by visible light.^30,31^

Although 3-terminal optoelectronic synaptic devices can effectively sense and process optical image information in nearly real time, demonstrating a considerable potential for ultrafast machine vision applications, they also show large-scale integration restrictions due to an increased pixel area. Such neuromorphic visual devices with a planar structure are unsuitable for applications such as self-driving cars and robots where the capture of stereodynamic images with a wide field of view with high resolution is imperative.^3^ As such, optoelectronic memristors with a two-terminal configuration can meet the criterion due to their smaller cell size, simpler device structure and fabrication process, and lower energy requirements.

While several studies on electrically controlled IGZO memristors have been reported, showcasing their great potential for both analog and digital resistive switching (RS) behaviors,^35−38^ the advantages of the optoelectronic memristor have not yet been fully investigated. A complete review of the available literature demonstrates that low photocurrent is one of the biggest limitations currently hampering further development of optoelectronic memristors in general.^13^ On IGZO-based optoelectronic memristors, only a few articles have been published to date. A UV-sensitive device was reported, using solution-processed IGZO, in which some of the synaptic functions could be realized, such as spike-time-dependent plasticity (STDP) and STM–LTM transition.^39^ Hu et al. proposed an interesting device based on an IGZO double layer in which a resistance decrease (Set) could be induced by blue light irradiation (420 nm) and a resistance increase (Reset) by red light (800 nm).^40^ More recently, in 2024, a heterostructure based on IGZO/tungsten oxide (WO_3–*x*_) was demonstrated with sensitivity up to 420 nm wavelength for image segmentation and object tracking.^41^ Although these studies demonstrate tremendous potential for IGZO optoelectronic memristors, they all report low *I*_light_/*I*_dark_ ratios (below 10) for all demonstrated wavelengths even after a few seconds of light irradiation, and none have explored the patterning of miniaturized devices, which should be critically considered. According to the review manuscript on the recommended methodology of RRAM studies published in 2019,^42^ it is evident that with a large device area, distinct RS properties and related physical mechanisms can emerge, potentially differing from those observed in miniaturized, patterned devices. Patterning is also fundamental for large-scale implementation of optoelectronic memristors, which, to our knowledge, has not been reported.

Here, we report a 4 μm^2^ optoelectronic memristor based on an H-doped IGZO layer, with visible range sensitivity for wavelengths up to 505 nm. Extremely high *I*_light_/*I*_dark_ ratios of 179, 93, and 12 are achieved for wavelengths of 365, 405, and 505 nm, respectively. This is explained by both the H-doping and by the use of a thin Ti/Au layer as the top electrode, which, despite its lower than 65% transmittance, increases the VO concentration in the IGZO layer, increasing photosensitivity. We also provide a comparative study on devices with different patterned areas, demonstrating the impact of device area on the *I*_light_/*I*_dark_ ratio and on the sensitivity to less energetic wavelengths, proving that miniaturization is a priority in this field. All significant synaptic functions are demonstrated such as STM–LTM transition, paired-pulse facilitation (PPF), STDP, and learning and forgetting capabilities, unraveling the potential of the device for integration with the IGZO-based display mature technology for neuromorphic vision sensors. The combination of light sensing and synaptic functionality at the pixel level opens up a myriad of possibilities for applications, such as adaptive brightness control, dynamic scene enhancement, and even image-processing tasks. Moreover, by reducing the system complexity and energy consumption, these neuromorphic display systems pave the way for immersive and adaptive display technologies that enhance the user experience across various domains, including augmented reality, smart signage, and human–machine interfaces.

## Results and Discussion

### Influence of the Top Contact on Optoelectronic Properties

**Figure 1:**
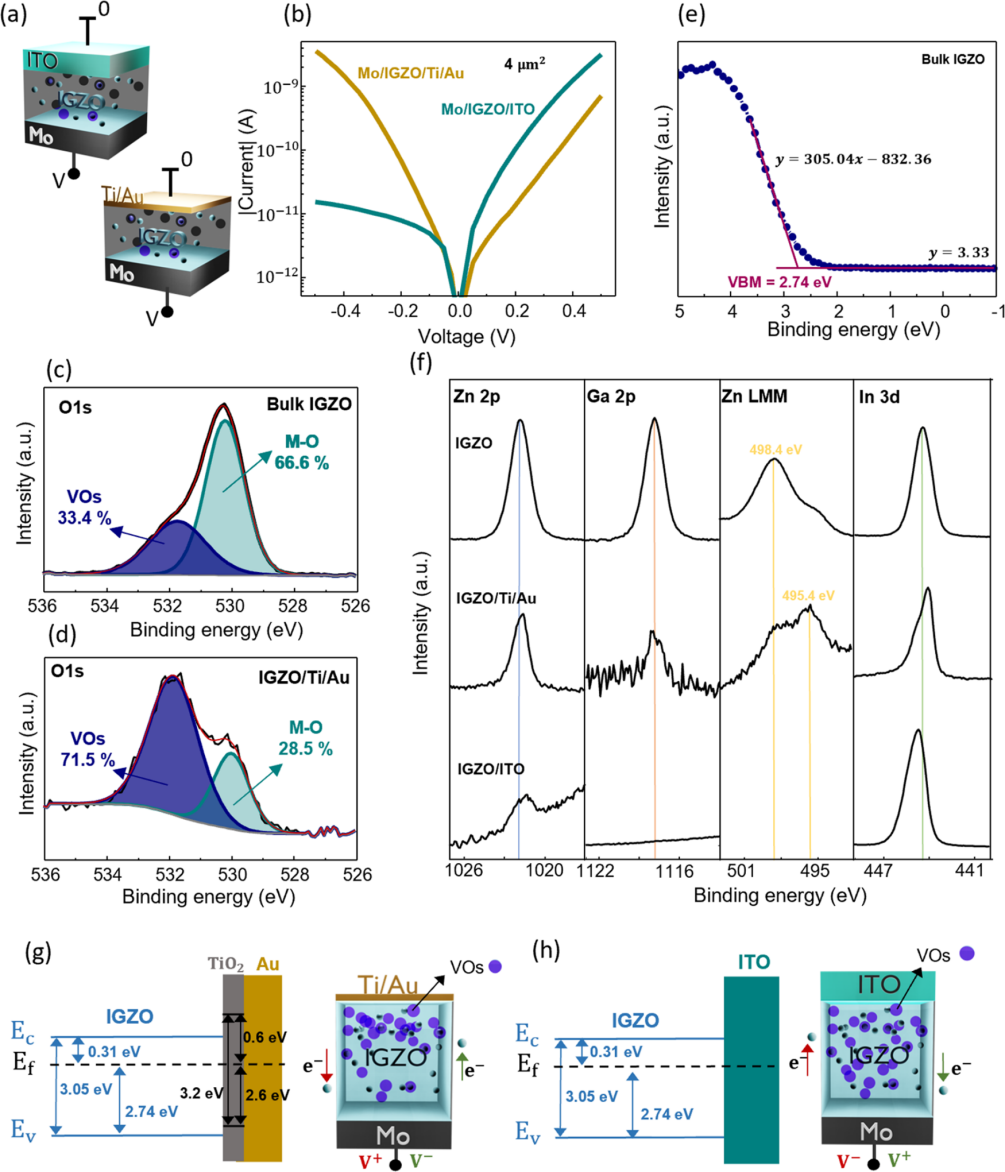
Analysis of IV characteristics in the dark. (a) Schematic illustration of the proposed IGZO devices, with Mo as the bottom contact and Ti/Au or ITO as the top contact. (b) IV characteristics of both pristine devices from −0.5 to 0.5 V, with voltage sweep applied to the bottom contact and ground at the top contact, displaying different rectifications for different top contacts. (c, d) Fitting of O 1s X-ray photoelectron spectra (XPS) displaying the oxygen vacancy (VO) percentage compared to the metal–oxygen (M–O) bonds for the IGZO bulk film and the IGZO/Ti/Au interface, respectively. (e) Valence band spectrum of the bulk IGZO film, used to calculate the valence band maxima (VBM). (f) Core level spectra of Zn 2p, Ga 2p, Zn LMM, and In 3d of IGZO and the Ti/Au and ITO interfaces displaying the offsets. (g, h) Schematic illustrations of the energy band diagram for the IGZO/Ti/Au and IGZO/ITO interfaces, respectively.

The undoped optoelectronic memristor structure is shown in Figure 1a. Molybdenum was used as the bottom contact, as it is often employed in thin-film transistors (TFTs) and would facilitate the targeted pixel integration.^16,43^ An oxygen plasma treatment was carried out on the Mo bottom contact to create a thin MoO_*x*_ layer, following our previous work.^35^ This oxide layer creates a barrier for electron injection and is usually the reason for the rectification found in the pristine devices. The photosensitive IGZO layer was then used as the active material, deposited by sputtering in an Ar/O_2_ atmosphere.^44^ Two different transparent top electrodes were investigated with the intent of comparing the electrical and optical device performance: indium-tin oxide (ITO) and thin titanium/gold (Ti/Au). ITO was deposited by sputtering with 65 nm thickness and presented a sheet resistance of 124 Ω/sq. Ti/Au was deposited by e-beam with 1 nm of Ti and 6 nm of Au. The atomic force microscopy (AFM) image of the Ti/Au film is shown in the Supporting Information (SI), Figure S1, with a very low root-mean-square (RMS) roughness of 402.2 pm. The absence of island formation confirms the morphological integrity of the top electrode. The devices were patterned via conventional photolithography into 4 μm^2^ devices. More details on the fabrication procedure can be found in the Experimental Section. In Figure 1b, the current–voltage (IV) characteristics, between −0.5 and 0.5 V, of both pristine devices are presented. Voltage is applied to the bottom Mo contact while keeping the top electrode (ITO or Ti/Au) grounded. A rectification ratio of more than 2 orders of magnitude is presented for the ITO configuration, with higher conductivity for positive polarities while the Ti/Au device presents a slight rectification in the opposite direction (higher conductivity for negative polarities).

In order to investigate the reason for the different rectifications, X-ray photoelectron spectroscopy (XPS) measurements were performed on the bulk IGZO film and the IGZO/ITO and Ti/Au interfaces. To ensure XPS detection of the IGZO cations at the interfaces, thin 1/3 nm Ti/Au and 4 nm ITO were deposited on the top of thick IGZO films on Si substrates. In Figure 1c,d, the O 1s spectra are shown for the bulk IGZO film and the IGZO/Ti/Au interface. The peaks were fitted with a Gaussian–Lorentzian function and the Shirley background subtraction. The O 1s spectra are deconvoluted into two peaks with binding energies of 530.1 and 531.8 eV, which are assigned to metal–oxygen bonds (M–O) and the oxygen from oxygen-poor regions, respectively. The oxygen deficiency increases drastically at the interface of IGZO/Ti/Au. In fact, the Ti oxygen getter effect has been reported several times.^45,46^ In this case, Ti reacts with the IGZO layer by removing oxygen ions and increasing the VO concentration at the interface. Titanium oxidation is confirmed by the Ti 2p_3/2_ binding energy at 458.8 eV (Figure S2). Note that the change to the O 1s emission cannot be related to the TiO_*x*_ formation because its lattice oxygen peak is at the same binding energy as that of IGZO.^47^ Unfortunately, the same analysis cannot be conducted at the IGZO/ITO interface because ITO itself contains a large fraction of oxygen. The Zn LMM Auger emission further reveals that the zinc in IGZO is reduced to a metallic state. The modified Auger parameter can be employed to determine the chemical state.^47^ The parameter was calculated by adding the binding energy of the Zn 2p_3/2_ peak and the kinetic energy (*E*_k_) of the Zn L3M45M45 Auger peak (Table 1). There is a clear reduction of Zn at the interface with Ti/Au due to the oxygen deficiency on IGZO found at the interface and the well-known Ti oxygen getter effect. For the IGZO/ITO interface, the Zn LMM Auger peak cannot be measured due to overlap with the Sn 3d emission.

**Table 1:** Modified Auger Parameter of the Bulk IGZO and IGZO/Ti/Au Interface

	*E*_b_ Zn 2p_3/2_ (eV)	*E*_k_ Zn LMM (eV)	M-Auger parameter	dominant species
IGZO	1021.85	988.2	2010.05	Zn(II) oxide
IGZO/Ti/Au	1021.55	991.2	2012.75	Zn(0)

The energy band alignment of the interfaces was also investigated by XPS. Figure 1e shows the valence band spectrum of the bulk IGZO film used to calculate the valence band maxima (VBM) of 2.74 eV determined by a linear extrapolation of the leading edge of the spectrum. As IGZO presents an optical band gap of 3.05 eV,^48^ the difference between the *E*_f_ and conduction band minimum (*E*_c_) was calculated to be 0.31 eV. The energy band alignment at the interface is then derived from the offsets of the core level spectra.^49,50^ The Zn 2p, Ga 2p, Zn LMM, and In 3d spectra of the IGZO bulk film and both studied interfaces (IGZO/Ti/Au and IGZO/ITO) are presented in Figure 1f. The core level Ga 2p emission of the IGZO/Ti/Au sample shows no binding energy shift. Because of the chemical reduction of zinc, the binding energy in the IGZO/Ti/Au sample cannot be used to analyze the energy band alignment at the interface. The Zn 2p emission in the IGZO/ITO sample shows a small shift to lower binding energies; however, the intensity is too low to be quantitative. These results indicate that at both interfaces, no depletion layers are formed in the IGZO, confirming the Ohmic contacts observed in the electrical characteristics.

Based on these results, a schematic illustration of the energy band diagrams of the IGZO/Ti/Au and IGZO/ITO interfaces shows flat bands in Figure 1g,h, respectively. For TiO_2_, the optical band gap and VBM values were extracted from elsewhere.^51^ In the current work, the reference electrode functions as the bottom contact for voltage application. Previously, we reported the presence of a rectifying barrier at the bottom interface of MoO_*x*_/IGZO.^35^ In the ITO device, an ohmic junction is also present at the top interface IGZO/ITO, and therefore, the rectification comes from the bottom interface, demonstrating properties consistent with our prior findings. However, in devices with a thin Ti/Au top electrode, Ti, which absorbs oxygen from the IGZO, becomes fully oxidized. This creates a TiO_2_/Au junction, which can therefore present a barrier to electron injection from Au.^52^ In this case, the slight rectification observed in this device can be explained by the second barrier arising from the Au/TiO_2_ interface.

**Figure 2:**
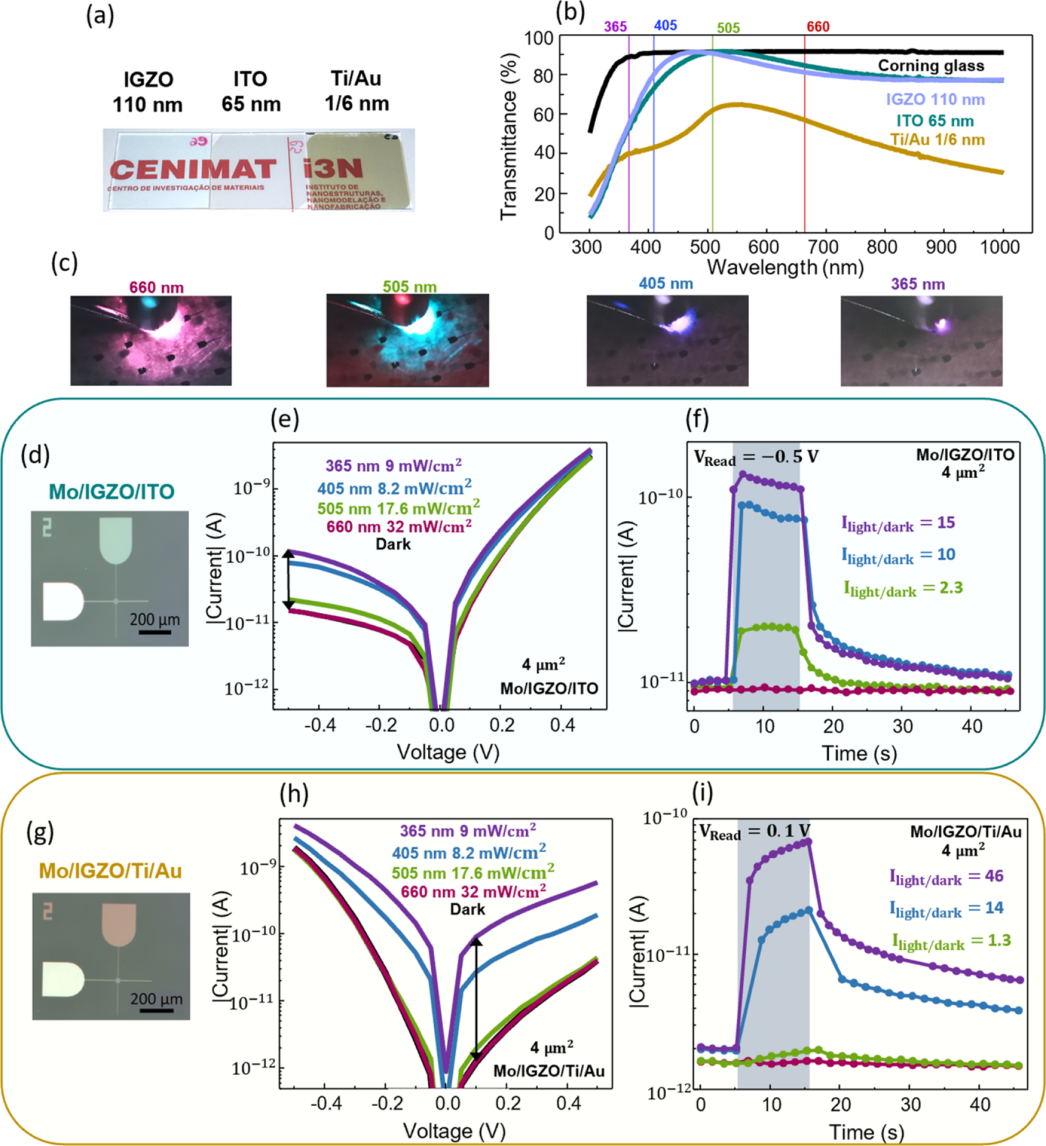
Optoelectronic properties of the IGZO-based memristor. (a, b) Image and transmittance results on IGZO, Ti/Au, and ITO films deposited on glass, respectively. (c) Images of a device being irradiated by the chosen light wavelengths. (d) Micrograph of the 4 μm^2^ ITO device with no intentional light input. (e) IV characteristics and (f) transient response with a *V*_Read_ of −0.5 V under 10 s of irradiation followed by 30 s in the dark of the ITO device with wavelengths of 660, 505, 405, and 365 nm. (g) Micrograph of the 4 μm^2^ Ti/Au device. (h) IV characteristics and (i) transient response with a *V*_Read_ of 0.1 V under 10 s of irradiation followed by 30 s in the dark of the Ti/Au device with wavelengths of 660, 505, 405, and 365 nm.

In the image displayed in Figure 2a, the transparency of IGZO, Ti/Au, and ITO can be separately evaluated. In Figure 2b, optical measurements reveal that IGZO and ITO have similar transmittance values, while Ti/Au transmittance does not surpass 65% for wavelengths ranging from 300 and 1000 nm. For the evaluation of the device response to light, light-emitting diodes (LEDs) with wavelengths of 660, 505, 405, and 365 nm were chosen for irradiation and placed on top of the devices, as presented in the images in Figure 2c. Figure 2d depicts a micrograph of a 4 μm^2^ device with ITO as the top contact. The optical response of the memristor can be evaluated from Figure 2e, where the IV characteristics are presented in the dark and under irradiation. Before any optical measurement, the devices underwent electrical Resets, which are clarified later in this paper, to eliminate any photoexcitation from ambient or microscope light. As explained before, under light stimulation, it is anticipated that neutral VOs on the IGZO are ionized and become positively charged (VO^+^ or VO^2+^). With a band gap of 3.05 eV, IGZO is expected to respond to UV light (3.1 eV) mostly,^48^ and even if the focus of this work is on visible light sensitivity, the UV performance is always shown throughout the paper for proper comparison with the state of the art. Accordingly, there seems to be no response to 660 nm red light, as the IV characteristics can be seen precisely on top of the measurements performed in the dark. The highest ratio between the measured current in the dark (*I*_dark_) and under light irradiation (*I*_light_) can be distinguished at −0.5 V for all other wavelengths, and this is, therefore, the read voltage (*V*_Read_) applied for the transient test presented in Figure 2f. Here, the current state is recorded for 10 s of constant light irradiation followed by 30 s in the dark. The lack of response to red light is confirmed and for the other wavelengths, *I*_light_/*I*_dark_ ratios of 15, 10, and 2.3 are achieved for 365, 405, and 505 nm irradiation, respectively. Photocurrent saturation is reached for all three wavelengths within 1 s of illumination, which means that the device is extremely fast. The PPC effect seems to undergo a fast decay, which is due to the high negative voltage applied. This voltage provides enough energy to accelerate the movement of electrons into the VOs, increasing the rate of neutralization of the photoexcited defects. It can be inferred then that, even if a *V*_Read_ of −0.5 V provides a higher *I*_light_/*I*_dark_ ratio, it also hinders the PPC effect and slowly decreases the photocurrent with increasing illumination time. Figure S3 displays the same test with a *V*_Read_ of 0.1 V, which shows the current state slightly increasing during irradiation. PPC decay is still quite fast, but this could be related to the low *I*_light_/*I*_dark_ ratio.

Similarly, the analysis of the device with Ti/Au as the top contact is presented in Figure 2g–i. The green response is very faint, as the Ti/Au has a transmittance of 61.5% at 505 nm. However, despite the even lower Ti/Au transmittance of 40.5% (at 365 nm UV) and 42.9% (at 405 nm blue), the *I*_light_/*I*_dark_ ratios achieved with this device are 46 and 14, respectively. This indicates that the benefits of using Ti/Au as the top electrode outweigh the losses derived from its lower transparency. A positive voltage of 0.1 V is applied as the *V*_Read_, and after 10 s of illumination, the photocurrent is still increasing, which means that it has not reached saturation. Compared with the results of the device with ITO as the top contact, it can be concluded that the Ti/Au device has a lower switching speed. This is easily explained by the lower transmittance of Ti/Au, which in turn results in a lower energy reaching the photosensitive layer IGZO. However, the PPC effect can be seen clearly with two different current levels retained after blue or UV irradiation.

It is important to note that the polarity of *V*_Read_ should be chosen for the lowest *I*_dark_ to provide the highest *I*_light_/*I*_dark_ ratios. For the ITO device, this means a negative *V*_Read_, and for Ti/Au, this means a positive *V*_Read_. The *I*_dark_ of the Ti/Au device is lower than the *I*_dark_ of the ITO device. However, *I*_light_ is comparable in both devices, despite the lower Ti/Au transmittance, because of the higher VO content at its top interface, which explains the superior performance of the Ti/Au devices.

### Optimization for Visible Light Detection by Hydrogen Doping

**Figure 3:**
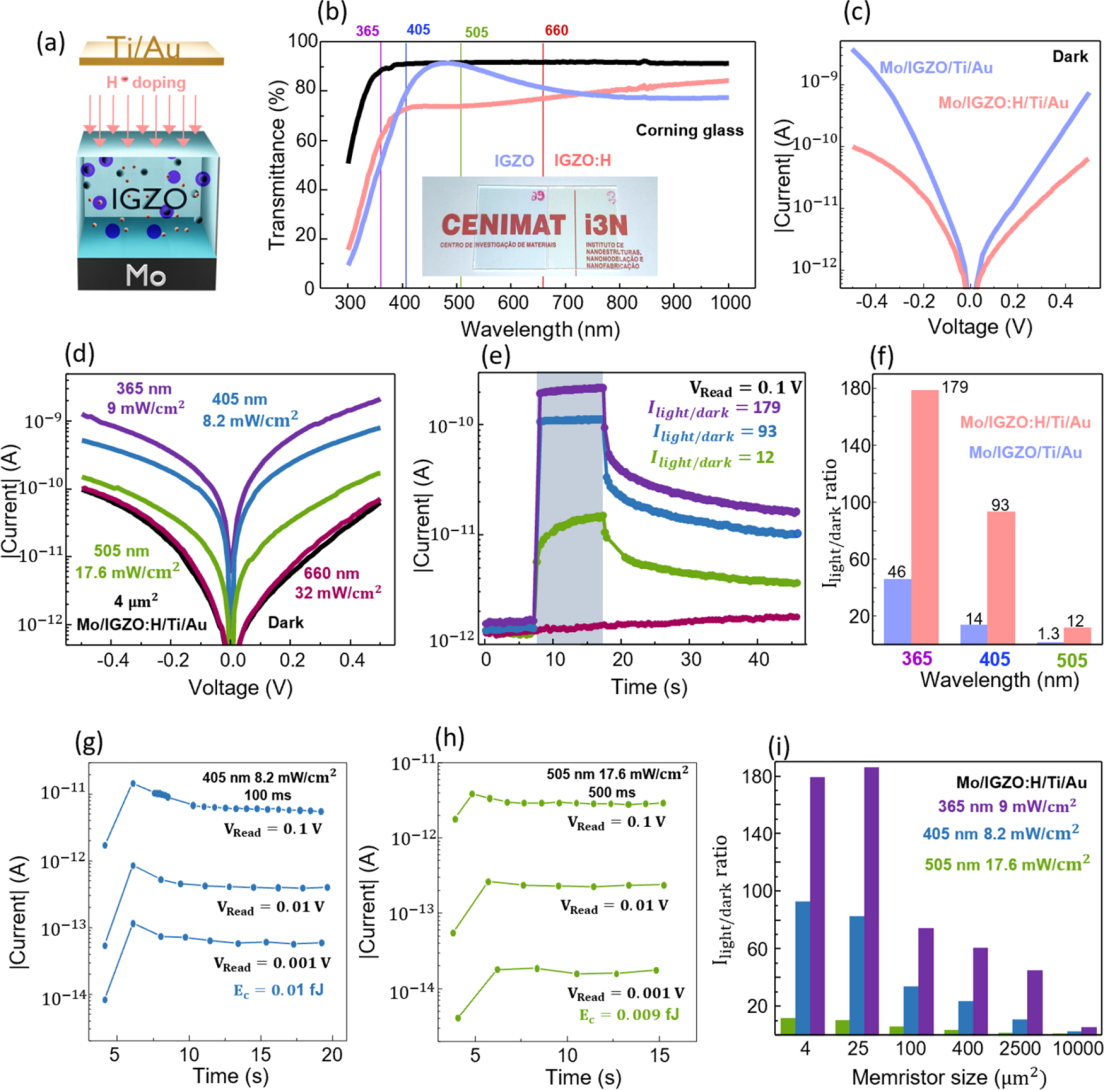
Optoelectronic properties of the hydrogen-doped (H-doped) IGZO memristor. (a) Schematic illustration of a H-doped IGZO memristor device. (b) Image and transmittance results of IGZO and H-doped IGZO films deposited on glass. (c) Comparison of the IV characteristics in the dark of the IGZO and the H-doped IGZO devices. (d) IV characteristics and (e) transient response with a *V*_Read_ of 0.1 V and under 10 s of irradiation followed by 30 s in the dark of the H-doped device for 660, 505, 405, and 365 nm illumination. (f) Comparison of the *I*_light_/*I*_dark_ ratios of both mentioned devices. (g) Photocurrent responses to a 100 ms pulse of blue light with *V*_Read_ values of 0.1, 0.01, and 0.001 V. (h) Photocurrent responses to a 500 ms pulse of green light with *V*_Read_ values of 0.1, 0.01, and 0.001 V. (i) Comparison of the *I*_light_/*I*_dark_ ratio of devices with different sizes.

To improve the visible range sensitivity, H-doping was performed on the IGZO film with both ITO and Ti/Au as the top contacts, as schematically illustrated in Figure 3a. In more detail, IGZO deposition was carried out in an Ar/O_2_/H_2_ atmosphere with 2% hydrogen gas. As can be seen in Figure 3b, the transmittance of the doped IGZO film is decreased in the green wavelength, indicating that the film absorbs more of the incident radiation than the undoped IGZO. In fact, even macroscopically, the doped film presents a greenish tone, while the undoped IGZO shows more of a blueish tonality. The atomic composition of the IGZO was calculated to be 2.2:1:1.1 from XPS measurements of the cation s orbitals (Ga 3s, Zn 3s, and In 4s) for both the doped and undoped films. The VO percentage was also not modified, as can be seen in Figure S4a. However, the valence band maximum was found to be 0.08 eV closer to the Fermi level in the IGZO:H films compared to undoped samples, as can be seen in Figure S5. With a constant band gap, this means that the doped films have 22 times lower carrier concentration than the undoped films (Boltzmann approximation). In Figure 3c, the IV characteristics of the pristine for the doped and undoped IGZO devices, with Ti/Au as the top contact, are compared. It is clear that by H-doping, the device has become less conductive overall. This can be explained by the lower carrier concentration in the doped film, which increased the series resistance of the IGZO:H layer.^53^

Similar to the undoped IGZO device, the VO percentage increases at the interface with Ti/Au, as can be confirmed from Figure S4b. It is known that H atoms can passivate VOs and create stable states in which H atoms become trapped at VOs (VO/2H).^54^ Moreover, in AOS, H atoms can also bond with oxygen, forming O–H bonds. Both of these processes increase subgap states, leading to improved optical absorption.^30,31^ By passivation of the defect states, hydrogen doping can also improve the mobility of the charge carriers. This allows for faster and more efficient transport of photogenerated carriers, leading to a higher photocurrent and quicker response times.^55,56^ This is confirmed by the significantly increased photocurrents of the doped memristor, presented in Figure 3d–f. In fact, for 10 s illumination, the *I*_light_/*I*_dark_ ratio increases from 1.3 to 12, from 14 to 93, and from 46 to 179 for wavelengths of 505, 405, and 365 nm, respectively. The photoresponsivity speed is also improved by H-doping, as saturation of the photocurrent is achieved much faster. Moreover, doping does not seem to increase the rate of PPC decay, which means that the device presents outstanding performance for SNN applications, namely, neuromorphic vision sensors.

For the H-doped device with ITO as the top contact, the same results could not be replicated, as shown in Figure S6. In the doped device, the rectification ratio significantly decreases and the conductance increases, with *I*_dark_ now more than 1 order of magnitude higher. *I*_light_ is also higher for all tested wavelengths; nonetheless, due to the increase in *I*_dark_, the *I*_light_/*I*_dark_ ratio is not improved with H-doping. One possible explanation is the fact that ITO is also strongly affected by H-doping. In fact, H-doping of ITO has been reported to enhance its conductivity.^57^ It is possible that there is an exchange of H atoms at the ITO/IGZO interface, increasing *I*_dark_. Another possible explanation is that the H-doping of the IGZO film decreases the barrier at the bottom interface MoO_*x*_/IGZO and, therefore, decreases the rectification ratio. This explanation is in line with previously reported findings of decreased contact resistance between Mo and IGZO by hydrogen plasma treatments.^58^

The switching speed of the optimized device was analyzed and is presented in Figure 3g,h for different *V*_Read_ values for 100 ms of blue light irradiation and 500 ms of green light illumination, respectively. Following a previously reported strategy,^59^ the energy consumption (*E*_c_) per single pulse was calculated through eq 1:
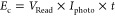
1

Therefore, for a *V*_Read_ of 0.001 V, *E*_c_ can be decreased to 0.01 fJ for blue and 0.009 fJ for green light, which is much lower compared to both biological synapses and to the state-of-the-art artificial photonic synapses for visible light detection, as summarized in Table S1. This shows that the proposed doped device is extremely power efficient, which is due to both the doping effect on the switching speed and the decreased patterned memristor area.

A comparison of the IV characteristics of 6 doped devices with different sizes in the dark is displayed in Figure S7, together with a full analysis of miniaturization. In summary, by decreasing the memristor area, the *I*_dark_ and *I*_light_ values are decreased and the *I*_light_/*I*_dark_ ratio is greatly enhanced, as shown in Figure 3i. As an example, by increasing the area of the device 100 times (400 μm^2^), the *I*_light_/*I*_dark_ ratio decreased from 179, 93, and 12 to 61, 24, and 3, respectively, for 10 s illumination with wavelengths of 505, 405, and 365 nm, respectively. This can be explained by the current density increase with downsizing, also observed for IGZO-based diodes,^60^ indicating that the current flow is not uniform across the entire device area, and in fact it flows through local current conduction paths.^61^

### Emulation of Synaptic Properties

**Figure 4:**
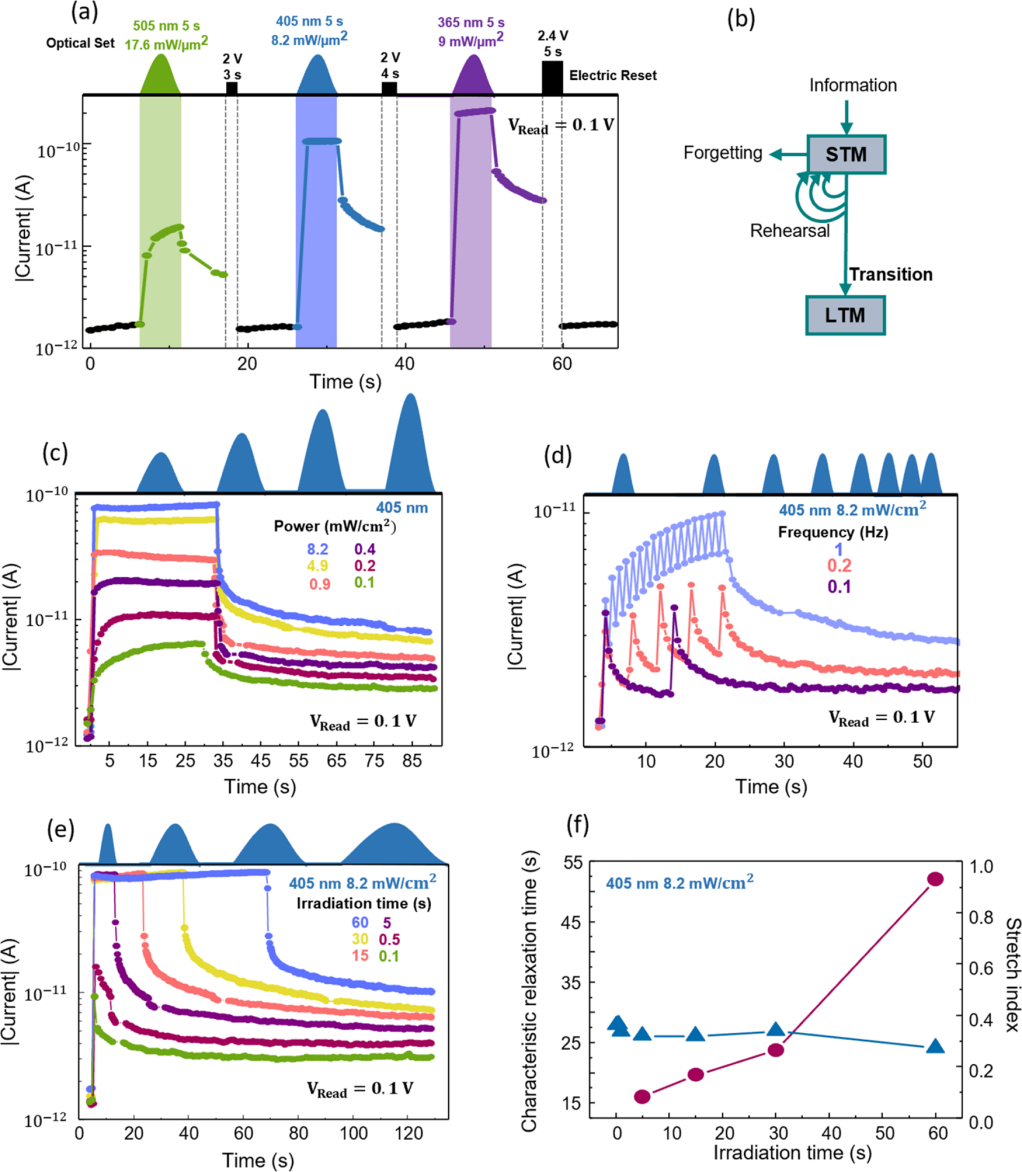
Reset by electrical pulse and state and PPC dependence on the power, time, and frequency of the optical input. (a) Increased conductance states achieved by decreased wavelength illumination and the respective electrical Reset pulses. (b) Schematic illustration of the transition from short-term memory (STM) to long-term memory (LTM). Different conductance states reached by (c) increased power, (d) increased frequency, and (e) increased time of illumination with a 405 nm wavelength LED. (f) Characteristic relaxation time and stretch index for different irradiation times.

Having developed a device (Mo/IGZO:H/Ti/Au) with such a potential for neuromorphic vision sensors, other figures of merit were investigated in this regard. First, the device should be fully controllable concerning Set (the increase of conductance by light) and Reset (the decrease of conductance by electrical pulse). In Figure 4a, different current states can be distinguished on irradiation with lights of different wavelengths. The higher the current state reached by light, the higher the voltage (or the longer the electrical pulse duration) required to perform a Reset and bring the current back to the value in the dark. The same test is shown in Figure S8 for the H-doped IGZO memristor with ITO as the top contact. Apart from the decreased *I*_light_/*I*_dark_ ratio, a similar behavior is observed. Low cycle-to-cycle (C2C) and device-to-device (D2D) variabilities are also crucial requirements for any envisioned application. In Figure S9, 10 cycles of UV, blue, and green illumination, followed by few seconds in the dark and a Reset pulse, are depicted with no considerable change in the device performance. Moreover, 10 devices with an area of 4 μm^2^ were measured in terms of their optical performance and *I*_dark_. The results are displayed in Figure S10 and show low variability. This minimum variability can be ascribed to the expected fabrication variability and the unavoidable randomness of the measurement in terms of LED positioning on top of the device. As physical vapor deposition tools that ensure good uniformity on large areas are used for all layers on the proposed structure, the batch-to-batch variability is also not expected to be significant.^62^

As previously discussed, the PPC decay on AOS can be used to emulate synaptic functions of the human brain. As such, this decay should be accurately controlled to simulate the transition between STM and LTM, as schematically illustrated in Figure 4b. According to the memory model suggested by Atkinson and Shiffrin, most of the information we receive is saved by the brain only temporarily, which is called STM. Subsequently, if the input is repeated several times, STM can transition to LTM. This behavior can be simulated by the H-doped IGZO optoelectronic memristor by increasing the power, illumination time, or frequency of the light input. Figure 4c–e show the impacts of these three parameters using blue radiation (405 nm). In Figure 4c, 6 different current states can be distinguished following 30 s of illumination and powers ranging from 0.1 to 8.2 mW/cm^2^, Figure 4d presents the PPC with varying frequency, from 0.1 to 1 Hz, and Figure 4e shows the effects of the illumination time on the photocurrent and PPC. The same measurements were performed for UV (365 nm) and green (505 nm) radiations, as presented in Figure S11. In all trials, different conductance states could be achieved by the varying conditions.

It is believed that the ionization of VOs (VO → VO^+^ + 1e^–^ or VO → VO^2+^ + 2e^–^) is responsible for the photocurrent observed in AOS. Following light irradiation, a recombination reaction takes place in which free electrons neutralize VOs (VO^+^ + 1e^–^ → VO or VO^2+^ + 2e^–^ → VO).^63^ Therefore, the manipulation of the PPC decay in AOS requires control of this reaction rate, which is directly related to the energy barrier necessary for the neutralization of ionized VOs. Therefore, while ionization is a rapid process occurring due to the optical energy provided to the system, neutralization is a thermally activated process with activation energy, which will take place gradually.^64^ IGZO is one of the AOS materials with a higher activation energy, which explains the prolonged PPC characteristics observed here. Additionally, it has been reported that higher activation energies can be achieved with lower wavelengths. This is related to a shift in the Fermi level to values closer to the conduction band minimum as a result of increased free electron concentration.^22^ A higher activation energy can, therefore, be achieved not only with lower wavelengths but also with a prolonged irradiation time or a more frequent input. In this work, the PPC decay was fitted following the Kohlrausch stretched exponential function, as it has been previously shown to well describe the transition from STM to LTM,
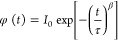
2in which φ(*t*) is the relaxation function, τ is the characteristic relaxation time, *I*_0_ is the current state immediately after light illumination, and β is the stretch index (0 < β < 1). The increase in the characteristic relaxation time implies a lower forgetting rate, demonstrating the transition from STM to LTM.^65^ It is important to note that the decay profile and characteristic relaxation time τ will be influenced by the initial carrier density, making it challenging to isolate the effects of recombination dynamics from the effects of the initial carrier density. Therefore, here, the fittings were performed on the decay of different irradiation times that had reached the same or very similar photocurrent (60, 30, 15, and 5 s) to ensure the same initial photogenerated carrier density that enables the accurate comparison of the recombination rate. The fittings can be found in Figure S12 for all of the tested wavelengths. In all cases, the relaxation time constant τ increases for longer irradiation time, as can be inferred from Figure 4f for blue light irradiation and Figure S12, corresponding to a clear transition from STM to LTM, meaning that we can effectively control the PPC behavior of the proposed device.

**Figure 5:**
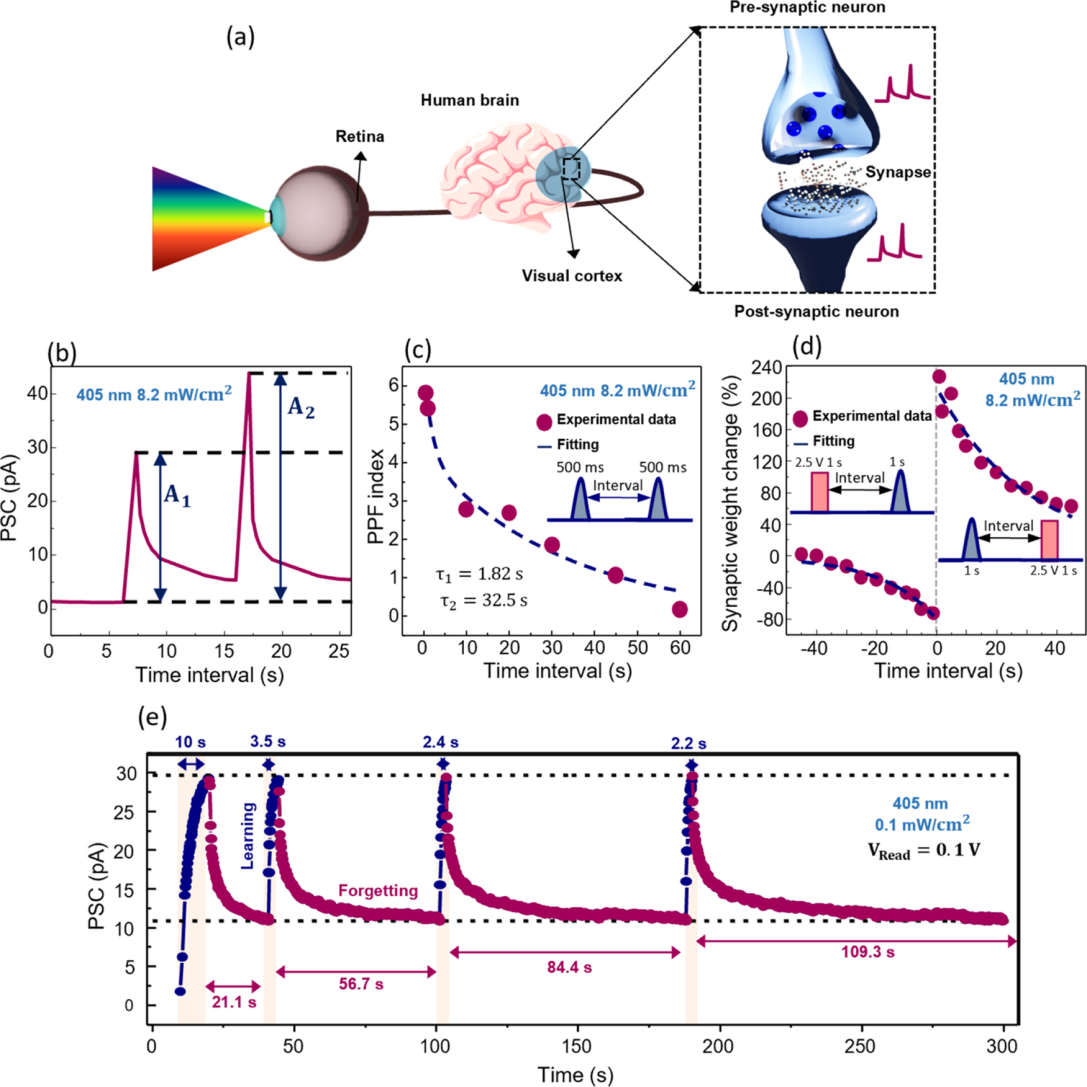
Synaptic functions emulated by the H-doped IGZO optoelectronic memristor. (a) Schematic illustration of the human visual system. (b) Postsynaptic current (PSC) evolution during a pair of optical pulses of 500 ms each with a time interval 10 s. (c) Paired-pulse facilitation (PPF) index for different time intervals. (d) STDP learning rule. An optical pulse of blue light for 1 s serves as the presynaptic pulse and an electrical pulse of −2.5 V for 1 s is implemented as the postsynaptic pulse. (e) Learning and Forgetting demonstration. Learning is performed by illumination with a 405 nm wavelength and 0.1 mW/cm^2^. Forgetting is performed in the dark by applying a *V*_Read_ of 0.1 V.

As illustrated in Figure 5a, the eyes collect information through light. This information is then processed in the visual cortex of the human brain by neurons and their synapses, involving the transmission of chemical or electrical spikes. Synapses are connected by a presynaptic neuron and a postsynaptic neuron. The connection between the two can be enhanced or weakened, which is known as a synaptic weight change and is responsible for mechanisms such as learning and memorizing. An artificial synapse should follow the learning rules of the human brain to accurately simulate it.

In a biological synapse, PPF is correlated to the synaptic weight becoming stronger after the application of two spikes in the presynaptic neuron. In other words, the postsynaptic current (PSC) triggered by the second pulse will increase the PSC caused by the first pulse due to the memory effect. The time interval between the two pulses (Δ*t*) determines the increase in the PPF index. The higher the Δ*t*, the lower the expected PPF index. In Figure 5b, the PSC evolution in the H-doped IGZO optoelectronic memristor, for a *V*_Read_ of 0.1 V, can be seen during a PPF test for two optical pulses with 500 ms each and a Δ*t* of 10 s. It is confirmed that the PSC is enhanced by the second spike. The PPF in the memristor is calculated by eq 3:
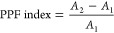
3in which *A*_2_ is the PSC after the second pulse and *A*_1_ is the PSC of the first pulse. The PPF index for increasing Δ*t* is given in Figure 5c. The experimental data were then fitted with eq 4:
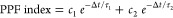
4where *c*_1_ and *c*_2_ are the initial facilitation magnitudes of different phases, and τ_1_ and τ_2_ are the characteristic relaxation times of the respective stages. In biological synapses, the decrease in the PPF decay with pulse interval can be ascribed to a transition between a fast memory stage of tens of milliseconds and a slower phase lasting hundreds of milliseconds.^7^ The memristor also presents this behavior, as can be seen by the fitting with eq 4 returning an τ_1_ of 1.82 s and an τ_2_ of 32.55 s.

Another important synaptic rule is STDP. According to STDP, the synaptic weight change (Δ*W*) is positive (connection becomes stronger) if a pulse applied to the presynaptic neuron arrives before a pulse is applied to the postsynaptic neuron. In contrast, if the postsynaptic pulse arrives before the presynaptic pulse, Δ*W* is negative (the connection becomes weaker). Moreover, Δ*W* should be weakened by noncoincidental neuronal firing.^66^ Here, Δ*t* represents the time interval between the pre- and postsynaptic pulses. Δ*W* represents the difference between the PSC after both spikes have been applied and the PSC before any pulse arrives. Therefore, the higher the Δ*t*, the lower the expected Δ*W*. To simulate this behavior with the H-doped IGZO optoelectronic memristor, the presynaptic and postsynaptic pulses were first decided as an optical pulse of 1 s (blue light) and an electrical pulse of 2.5 V for 1 s applied to the bottom contact, respectively. The results are displayed in Figure 5d in which the Δ*W* is positive for Δ*t* > 0 and negative for Δ*t* < 0. Moreover, a decrease in |Δ*W*| is also observed for Δ*t* ≫ 0 and Δ*t* ≪ 0. The STDP learning rule was, therefore, successfully replicated in our optoelectronic synapse. The STDP time window was fitted by eq 5:
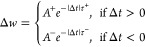
5where *A*^+^, *A*^–^, τ^+^, and τ^–^ are free parameters representing the scaling factor and time constant of the exponential function, respectively, which can be discovered by fitting the experimental data with no restriction.^66^ The STDP data are well described using this equation, returning a τ^+^ of 32 ms and a τ^–^ of 54 ms. It is important to note that the STDP characteristics present a typical asymmetric Hebbian learning rule form, similar to a hippocampal neuron culture,^67^ as often shown in previous studies reported on synaptic devices.^68^ Here, as the effect caused by the presynaptic pulse (optical) does not correspond to the one caused by the postsynaptic pulse (electrical), the synaptic weight changes are not symmetric in Δ*t* > 0 and Δ*t* < 0. The higher current change achieved in a positive time interval could arise from more significant Joule heating, which can enhance mobility and accelerate the switching process. This can be adjusted by the calibration of different pulse schemes (duration and amplitude), the waveform shape (rise and fall time), or even the application of a current limiting technique. As other studies report, the STDP characteristics can be tuned by the development of appropriate spiking inputs and order depending on the envisioned application.^69^

Finally, learning and forgetting behaviors were also reproduced by our memristor. In Figure 5e, 4 cycles of learning and forgetting processes are shown. Learning is simulated by applying a constant blue light with a 0.1 mW/cm^2^ intensity. Each learning cycle is stopped as soon as the maximum current achieved in the first learning is reached. Forgetting is performed in the dark by applying solely a *V*_Read_ of 0.1 V. Each forgetting cycle is stopped as soon as the current state from the first forgetting process is reached. The time it takes the device to learn decreases with each cycle, starting with 10 s in the first cycle and finishing with 2.2 s at the fourth cycle, indicating faster relearning with task repetition. In contrast, the forgetting time increases with each cycle, starting with 21.1 s in the first cycle and finishing with 109.3 s in the fourth cycle, indicating harder forgetting, once again successfully mimicking the human brain. In Figure S13, the learning and forgetting behavior is shown in a different form and by pulsed light.

## Conclusions

In summary, a 4 μm^2^ optoelectronic memristor based on IGZO is presented in this work with enhanced characteristics. Thin Ti/Au is employed as a transparent top electrode as it increases the VO concentration at the IGZO interface due to the oxygen affinity of Ti, improving the optical performance. H-doping is performed by introducing H_2_ in the IGZO sputtering deposition to induce the creation of subgap states for visible range photodetection. Extremely high light/dark ratios of 179, 93, and 12 are observed for UV, blue, and green illumination, respectively. Moreover, all relevant synaptic functions for SNNs are demonstrated, such as STM–LTM transition, PPF, STDP, and learning and forgetting capabilities by manipulating the PPC decay. Therefore, the proposed optoelectronic memristor can be considered for applications in flexible neuromorphic vision sensors, representing an enormous enhancement of conventional artificial visual systems and in more detail to the IGZO-based display AOS pixel-driver circuit technology employed in commercial IGZO flat-panel displays.

## Experimental Section

The devices were fabricated on Corning Eagle glass, previously cleaned in repeated ultrasonic baths of acetone and isopropanol, and rinsed with deionized water and dry nitrogen.

For the bottom electrode of the optoelectronic memristors, radio frequency (RF) magnetron sputtering was performed to deposit a 70 nm thick Mo layer in an AJA ATC-1800 system with a flow rate of 50 sccm of Ar, a sputtering power of 175 W (3 in. target), and a deposition pressure of 1.7 mTorr. Then, reactive ion etching in a Trion Phantom 3 system was used with SF_6_ to pattern this layer.

Then, oxygen plasma treatment was carried out on the bottom electrodes inside the sputtering chamber before IGZO deposition. The parameters used were a flow rate of 20 sccm of oxygen gas with 10 W of substrate bias and an RF power to the Ga_2_O_3_ target of 40 W to create the plasma for 10 min under 20 mTorr pressure. 110 nm of IGZO were deposited by RF magnetron cosputtering from three ceramic oxide targets. The sputtering powers used on each target (all 2″ diameter) were In_2_O_3_ 121 W, Ga_2_O_3_ 100 W, and ZnO 50 W using a flow rate of 20 sccm of Ar and 5 sccm of O_2_ for the standard IGZO film and 14 sccms of Ar, 3 sccms of O_2_, and 0.3 sccms of H_2_ for the hydrogen-doped IGZO film. The deposition pressure was kept constant at 2.3 mTorr. The In:Ga:Zn atomic composition of the deposited films was 2.2:1.0:1.1 for a normalized Ga concentration. The IGZO compositions were estimated by X-ray photoelectron spectroscopy (XPS) results through the area report on the Ga 3s, Zn 3s, and In 4s spectra.

A lift-off procedure was employed to pattern the IGZO and the top electrodes. A thin 1 nm layer of Ti was deposited immediately followed by the evaporation of 6 nm of Au by e-beam evaporation in a homemade apparatus, without breaking the vacuum. For the devices with ITO as the top electrode, a 65 nm thick ITO layer was deposited in an AJA ATC-1800 system using a single target (2″ diameter, 90:10 wt %) with a flow rate of 20 sccm of Ar and 0.25 sccm of O_2_, a sputtering power of 95 W, and a deposition pressure of 1.2 mTorr. All depositions were carried out with no intentional heating, and no annealing steps were performed.

A UV–vis–NIR spectrophotometer, PerkinElmer Lambda 950, was used to acquire the transmittance of the various transparent films between 1000 and 299.5 nm with 1.5 nm steps. The sheet resistances of the films were measured by the four-point probe method.

The electrical characterization of the optoelectronic memristors was conducted using a Keithley 4200 SCS semiconductor analyzer connected to a Janis ST-500 probe station. The DC sweeps and transient responses were acquired by applying a read voltage (*V*_Read_) to the bottom electrode while maintaining the top electrode connected to the ground. Fiber-coupled LEDs of 660, 505, 405, and 365 nm wavelengths from Thorlabs were then placed on top of each measuring device by an optical arm, a part of the Janis probe station. These LEDs were connected to an LED driver from Thorlabs that allows the accurate application of pulses from 1 Hz onward. For clarification of the setup, Supporting Information, Video S1 can be viewed.

XPS measurements were performed with a Kratos Axis Supra instrument, using a monochromatic Al Kα source running at 150 W. The analyzer was set to a pass energy of 10 eV for detail scans and 80 eV for surveys. CasaXPS version 2.3.25PR1.0 was used for data analysis. The Fermi level was calibrated by using a sputter-cleaned gold sample. AFM topographs were acquired with an Asylum Research MFP-3D Standalone system (Oxford Instruments, U.K.) operated in tapping mode under ambient room conditions. Commercially available silicon probes were used (Olympus AC160TS, Olympus Corporation, Japan; *f*_0_ = 300 kHz, *k* = 29 N/m), and the resulting topographs were exported using Asylum Research’s software packages after low-level flattening.

## Supporting Information Available

In summary, the Ti 2p XPS spectra from the IGZO/Ti/Au interface are presented to prove the oxidation of Ti by extracting O ions from IGZO; the O 1s spectra are also shown for a direct comparison of the VO quantity in the bulk doped IGZO film and the doped IGZO/Ti/Au interface; on the ITO top electrode, additional results are presented, namely, the transient response showing photocurrent under a *V*_Read_ of 0.1 V, *I*–*t* showing the photoresponse of the device with doped and undoped IGZO, and the electrical Reset operation; on the optimized device with IGZO:H and Ti/Au as the top electrode, the studies on device size, C2C, and D2D variability are provided; finally, different current states are shown to be achieved by increasing the light power, irradiation time, and pulse frequency for green and UV lights; transitions from STM to LTM are shown with increasing irradiation time by fittings with the Kohlrausch stretched exponential function; a table is also provided for a comparison of the device size and energy consumption of the proposed optimized device with the state-of-the-art synaptic optoelectronic devices for visible light detection (PDF)
Video S1 (MP4)
