# Supplementary material for: Unlocking Neuromorphic Vision: Advancements in IGZO-Based Optoelectronic Memristors with Visible Range Sensitivity
Source: ACS Appl Electron Mater. 2024 Jul 5;6(7):5230–43. doi: 10.1021/acsaelm.4c00752 (PMC11270833; doi:10.1021/acsaelm.4c00752)
Supplement: Supplementary file 1 — el4c00752_si_001.pdf [file el4c00752_si_001.pdf]

Supporting Information

Unlocking Neuromorphic Vision: Advancements in IGZO-based Optoelectronic Memristors with Visible Range Sensitivity

Maria Elias Pereira[1], Jonas Deuermeier[1], Rodrigo Martins[1], Pedro Barquinha[1] and Asal Kiazadeh[1]***

[1] i3N/CENIMAT, Department of Materials Science, NOVA School of Science and Technology and CEMOP/UNINOVA, NOVA University Lisbon, Campus de Caparica, 2829-516 Caparica, Portugal

*mel.pereira@campus.fct.unl.pt **a.kiazadeh@fct.unl.pt

Keywords: IGZO optoelectronic synapse; IGZO memristor; Hydrogen-doping; Visible range detection; Spiking neural networks; Neuromorphic vision sensors.

In Figure S1, the atomic force microscope (AFM) image of the Ti/Au film with 7 nm is presented showing low root mean square (RMS) roughness of 402.2 pm.

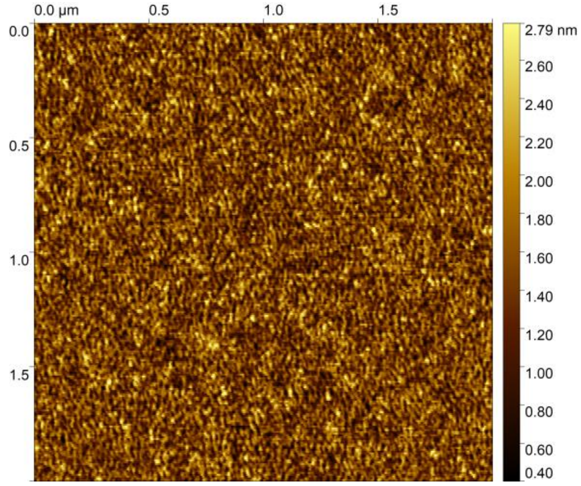

Figure S 1. AFM image of Ti/Au surface.

In Figure S2, the Ti $2p_{3/2}$ XPS spectra is shown. The titanium oxidation is confirmed by the Ti $2p_{3/2}$ binding energy at 458.8 eV. Ti reacts with the IGZO layer by removing oxygen ions increasing VOs concentration at the interface.

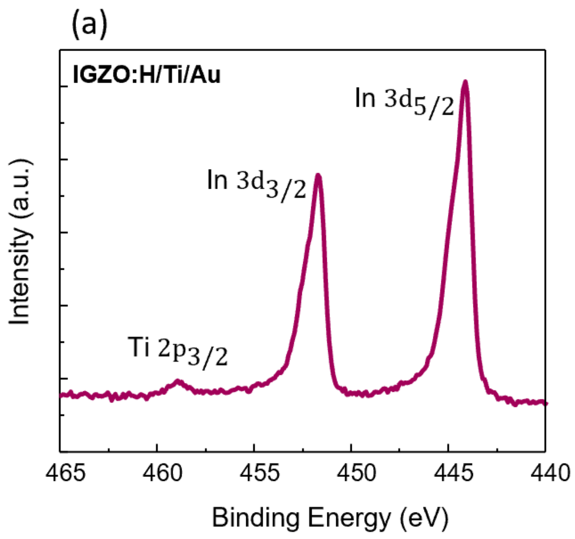

Figure S 2. Ti 2p XPS spectra.

In Figure S3, the transient response for 10 s of illumination, followed by 30 s in the dark, of the Mo/IGZO/ITO device with a V_{Read} of 0.1 V is presented. There is no response to red light. For the other wavelengths, I_{light}/I_{dark} ratios of 2.8, 2.3 and 1.2 are achieved for 365, 405 and 505 nm irradiation, respectively. Higher I_{light}/I_{dark} ratios are accomplished for a V_{Read} of -0.5 V, as discussed in the main article. However, the higher voltage applied forces electrons into VOs decreasing the photocurrent during illumination and fastening the PPC decay. For a V_{Read} of 0.1 V this is solved, and the photocurrent continues to slightly increase during illumination. The reason why the photocurrent does not increase during irradiation as much as in the Ti/Au devices lies in the transmittance of the top electrodes. Since ITO is more transparent than the Ti/Au, the light intensity reaching the IGZO layer is higher and therefore the photoresponse is faster. The PPC decay is still fast, but this could be due to the very low I_{light}/I_{dark} ratios. Moreover, the I_{dark} is more than 1 order of magnitude higher for the ITO device than for the Ti/Au device for the same V_{Read}.

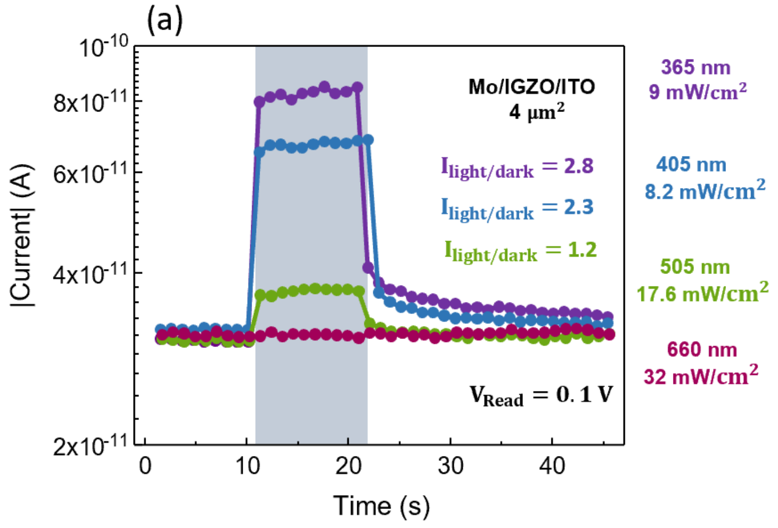

Figure S 3. Transient response of the Mo/IGZO/ITO device for 10 s of illumination followed by 30 s in the dark with V_{Read} of 0.1 V for different wavelengths.

In Figure S4, the O 1s spectra is shown for the bulk hydrogen-doped (H-doped) IGZO film and the H-doped IGZO/Ti/Au interface. The peaks were fitted with a Gaussian-Lorentzian (G-L) function and a Shirley background subtraction. Similarly to the undoped IGZO results presented in Figure 1 of the main paper, the VOs percentage increases drastically from 34.6% to 67.7% at the interface with Ti/Au. This result confirms the Ti oxygen getter effect.

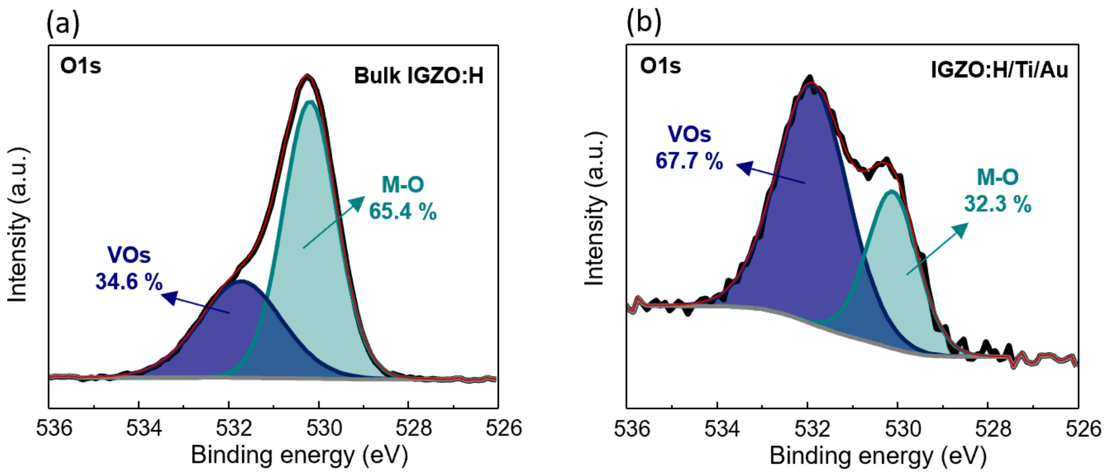

Figure S 4. (a) and (b) Fitting of O 1s XPS spectra displaying the oxygen vacancies (VOs) percentage compared to the metal-oxygen (M-O) bonds percentage for the doped-IGZO bulk film and the doped-IGZO/Ti/Au interface, respectively.

In Figure S5, the valence band spectrum of the H-doped IGZO film is presented, used to calculate the valence band maxima (VBM) of 2.66 eV determined by linear extrapolation of the leading edge of the spectrum. This presents a decrease of 0.08 eV compared to the undoped IGZO film, which means the carrier concentration has decreased 22 times with the doping (Boltzmann approximation).

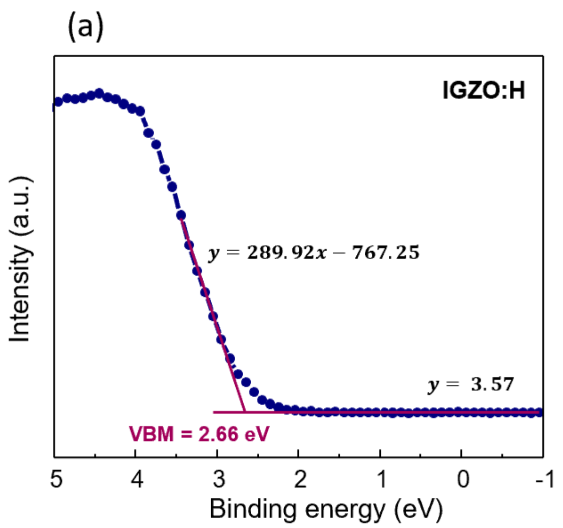

Figure S 5. Valence band spectra of the bulk IGZO:H film, used to calculate the valence band maxima (VBM).

In Figure S6, the H-doped IGZO device with ITO as top contact is analysed. Figure S5(a) shows the IV characteristic of the IV characteristic in the dark, between -0.5 and 0.5 V, of the undoped device and the doped device. The conductance state is substantially increased, especially for negative polarities which means the I_{dark} is increased with doping. This can be related to H diffusion to the ITO which would increase the conductance of the top contact. In Figure S5(b), the transient response of the optoelectronic memristor can be found for 365, 405 and 505 nm wavelength irradiation. I_{light}/I_{dark} ratios of 11.6, 6.5 and 2.2 can be distinguished, respectively, which is not an improvement in relation to the undoped-device.

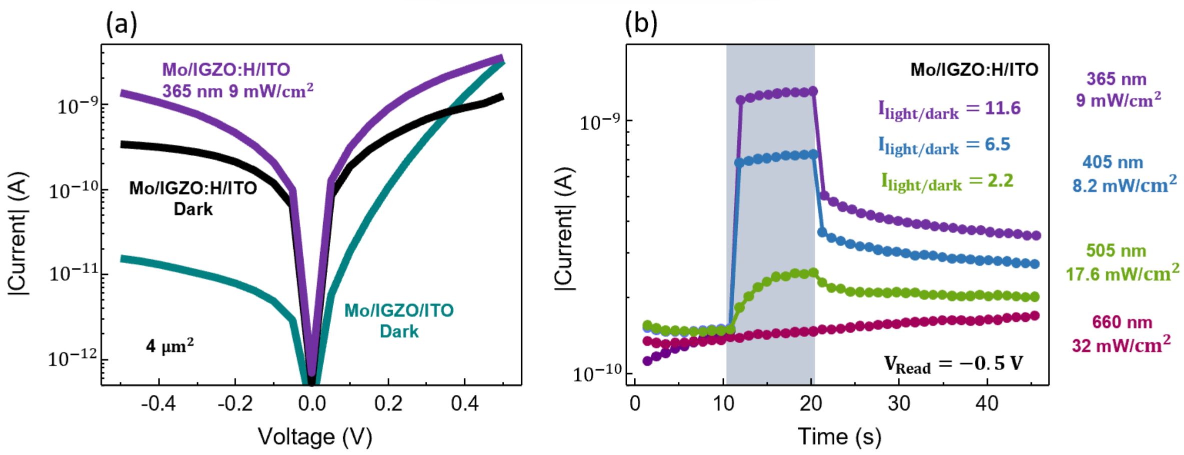

Figure S 6. (a) Comparison of the IV characteristic in the dark of the IGZO and the H-doped IGZO devices, with ITO as top contact. (b) Transient response with V_{Read} of -0.5 V and 10 s of irradiation followed by 30 s in the dark of the H-doped device with ITO as top contact for 660, 505, 405 and 365 nm illumination.

In Figure S7, H-doped with Ti/Au as top contact devices with different areas are analysed. The IV characteristic comparison in the dark can be consulted in S7(a) for devices with 4, 25, 100, 400, 2500 and 10000 µm^2. In Figure S7(b), the summary of the I_{dark} and I_{light} for 505, 405 and 365 nm wavelength illumination is presented in regard to the transient results shown in Figure S7(c). A steady increase of all mentioned currents is noted with increasing memristor size. However, the I_{light}/I_{dark} ratio is greatly reduced. Green sensitivity is also gradually lost as the memristor size increases. In fact, the 10000 µm^2 device shows no green detection and reduced ratios of 5 and 3 for UV and blue illumination, respectively.

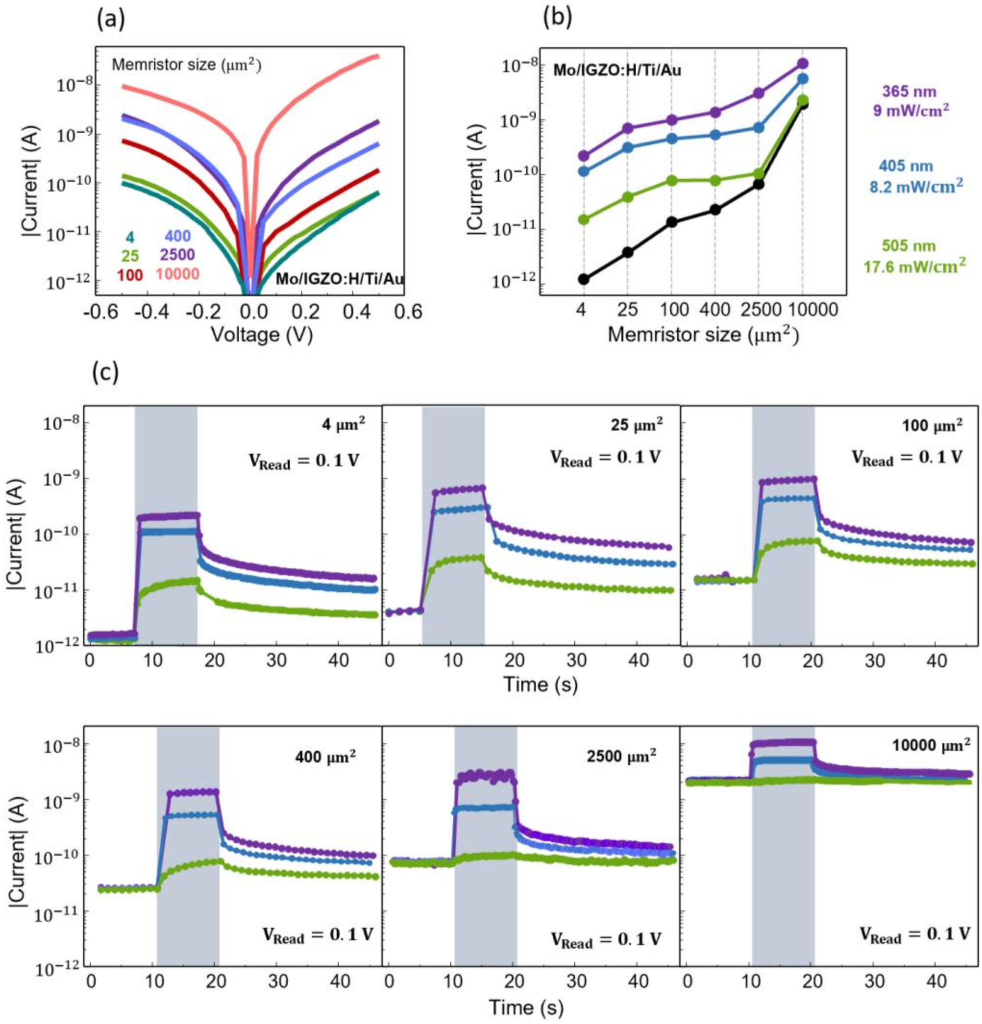

Figure S 7. (a) IV characteristic in the dark from -0.5 to 0.5 V for Mo/IGZO:H/Ti/Au devices with different sizes. (b) I_{dark} and photocurrents after 10 s of illumination with 365, 405 and 505 nm wavelength for devices with different sizes and (c) Transient response of devices with different sizes for 10 s of illumination followed by 30 s in the dark with V_{Read} of 0.1 V for different wavelengths.

In Figure S8, the transient response to optical and electrical inputs on the doped memristor with ITO as top contact can be evaluated. Increasing current states are achieved with more energetic wavelengths. The Reset electrical pulses are also discriminated. The higher the current state, the higher the voltage or the longer the pulse required to perform a full Reset.

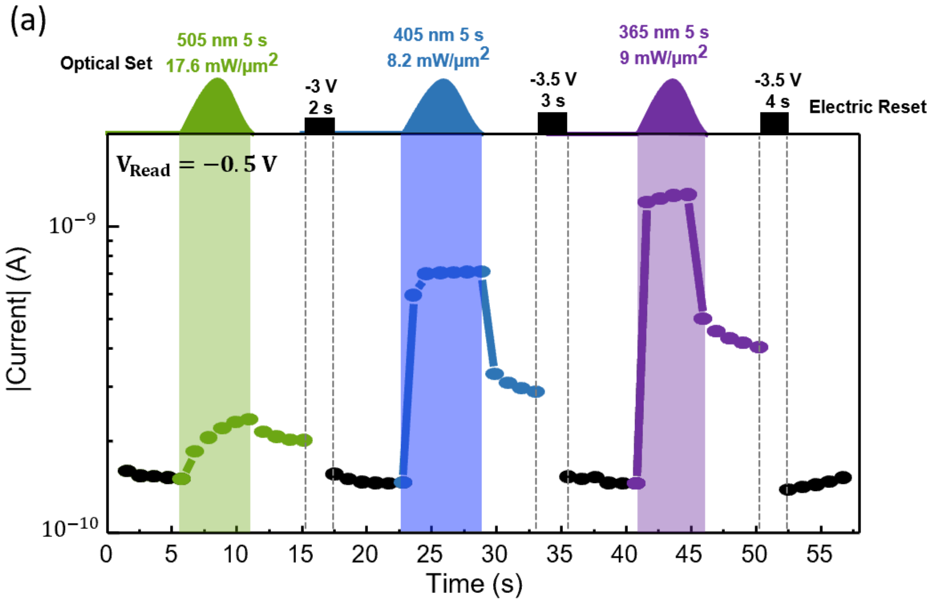

Figure S 8. (a) Increased conductance states achieved by decreased wavelengths illumination and respective electrical Reset pulse for the Mo/IGZO:H/ITO device.

Cycle-to-cycle (C2C) variability evaluation was performed on the optimized devices with H-doping and Ti/Au as top contact. The results can be found in Figure S9, where the transient response to 10 cycles of UV, blue and green illumination, followed by few seconds in the dark and a Reset pulse, is presented with no considerable change in device performance.

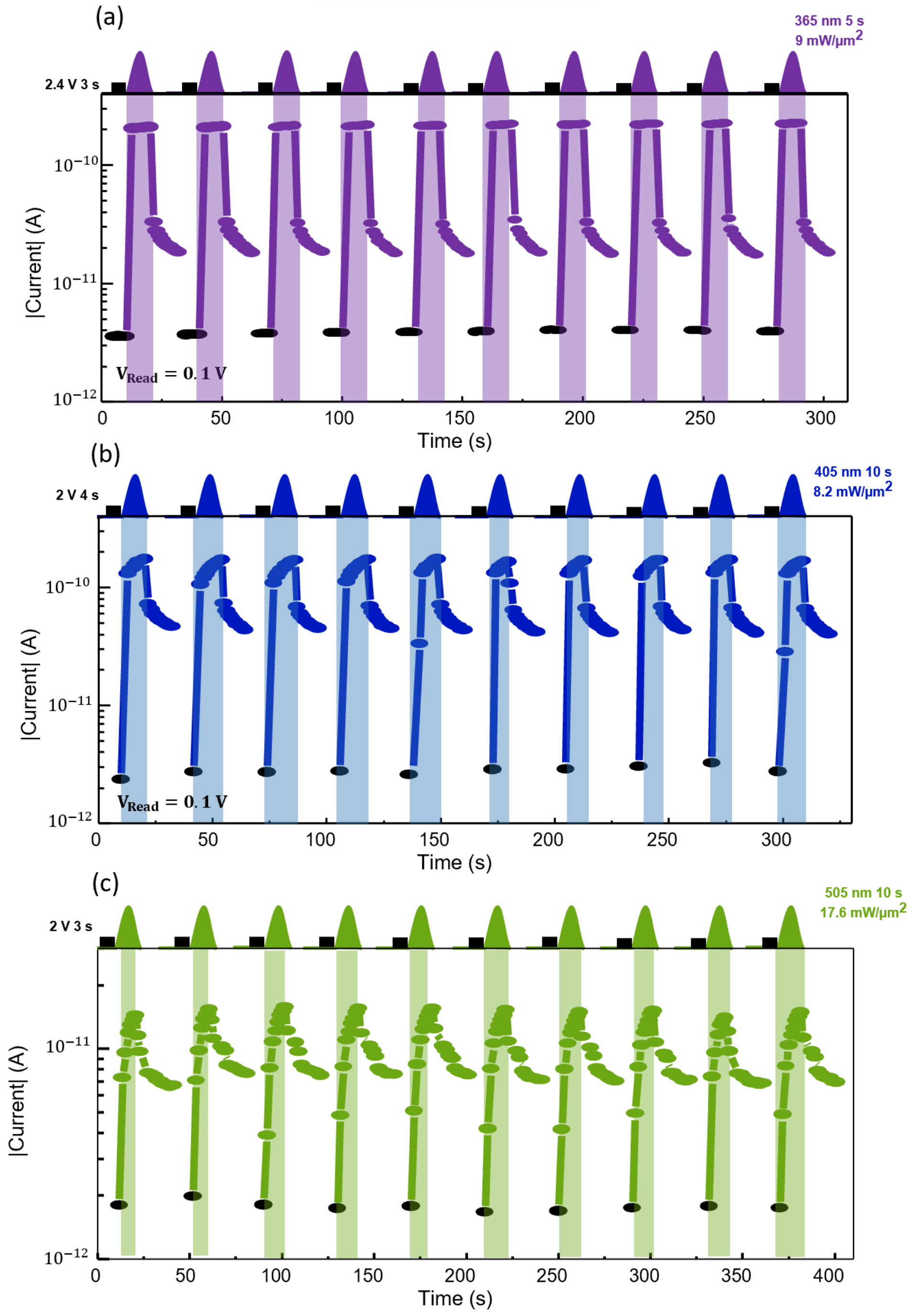

Figure S 9. 10 cycles of (a) UV, (b) blue and (c) green illumination, followed by a few seconds in the dark and a Reset pulse for the Mo/IGZO:H/Ti/Au device.

Device to device variability (D2D) was evaluated by the measurement of 10 devices with area of 4 μm^2 of their optical performance and I_{dark}. The results are displayed in Figure S10 and show minimal variability. Moreover, the variability that is presented is due to the unavoidable randomness introduced in the measurement by human LED positioning, on top of the device.

(a)

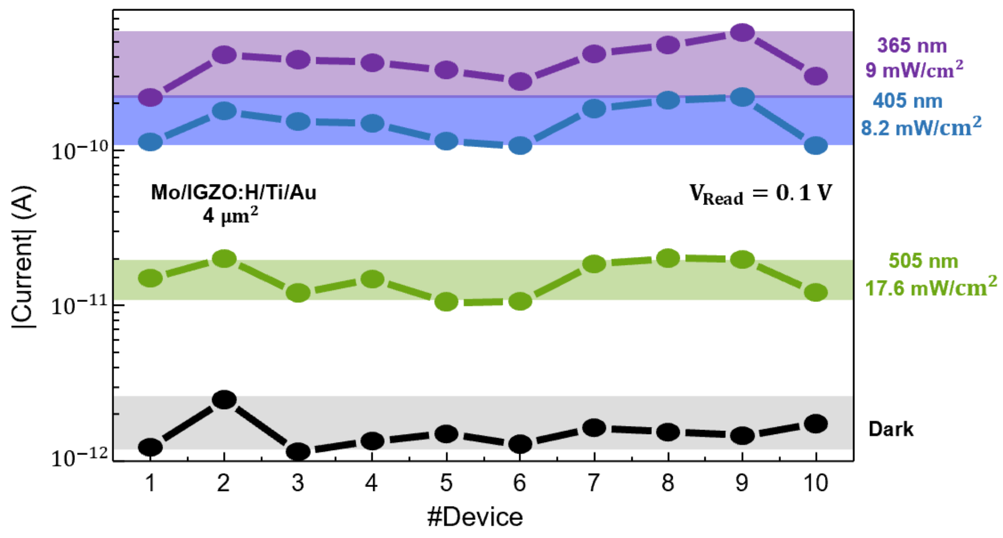

Figure S 10. (a) I_{dark} and photocurrents after 10 s of illumination with 365, 405 and 505 nm wavelengths for 10 randomly chosen 4 μm^2 devices.

In Figure S11, the effective manipulation of the photocurrent and PPC is shown by increasing power, illumination time or frequency of the light input. S11(a), S11(b) and S11(c) presents the results for optical green inputs (505 nm wavelength) and S11(d), S11(e) and S11(f) are in regard to optical UV inputs (365 nm wavelength).

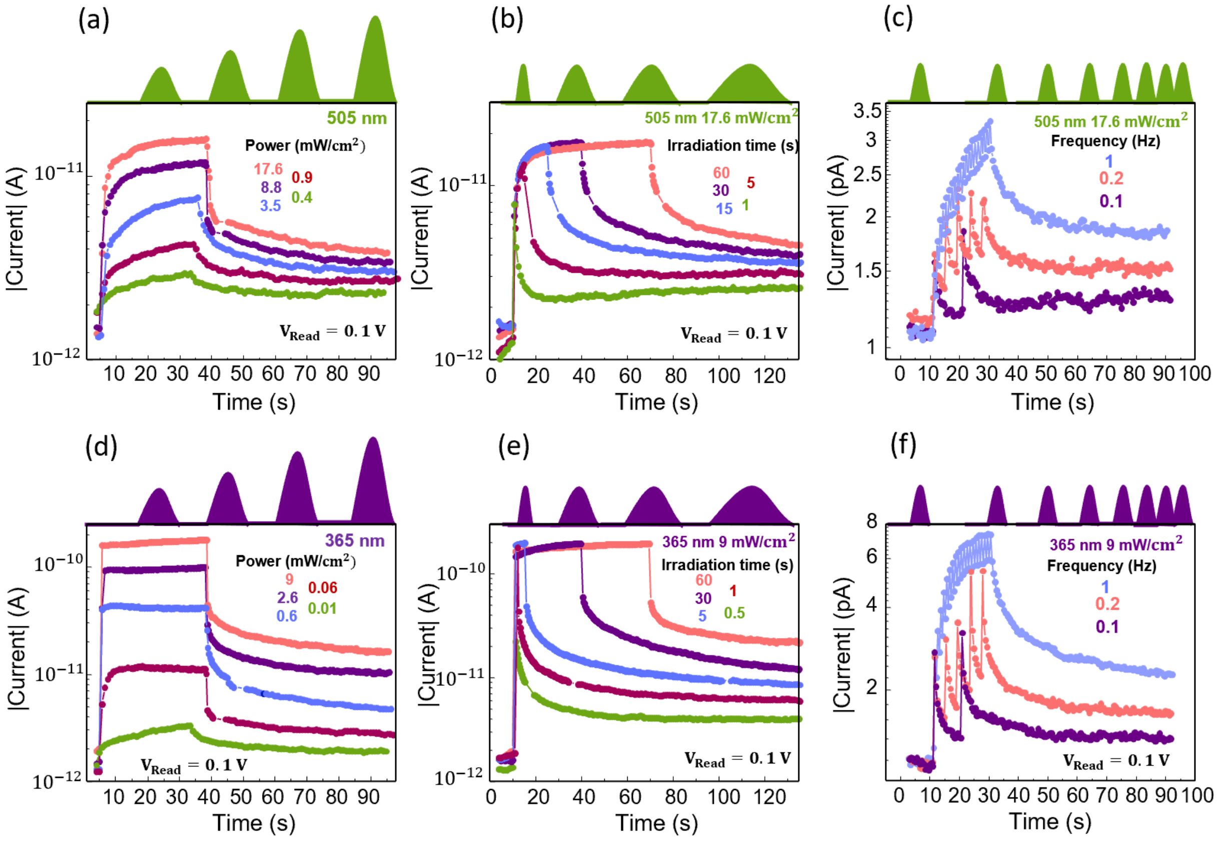

Figure S 11. Different conductance states reached by (a) increased power, (b) increased time and (c) increased frequency of illumination with a 505 nm wavelength LED. Different conductance states reached by (d) increased power, (e) increased time and (f) increased frequency of illumination with a 365 nm wavelength LED.

In Figure S12, the Kohlrausch stretched exponential fitting (equation 2 of the main paper) on the PPC that follows different irradiation times is shown for the tested wavelengths- Figure S12(a)- 405 nm, Figure S12(b)- 505 nm and Figure S12(c)- 365 nm. The same photocurrent is reached in each of the different irradiation times assuring an accurate comparison of the recombination reaction. It can be seen that the PPC decay is effectively manipulated by increased exposure simulating task repetition. In Figure S12(d) and S12(e), the characteristic relaxation time and stretch index are presented for different (d) 505 nm and (e) 365 nm illumination times that further confirm that the PPC decay is slower for longer irradiation times.

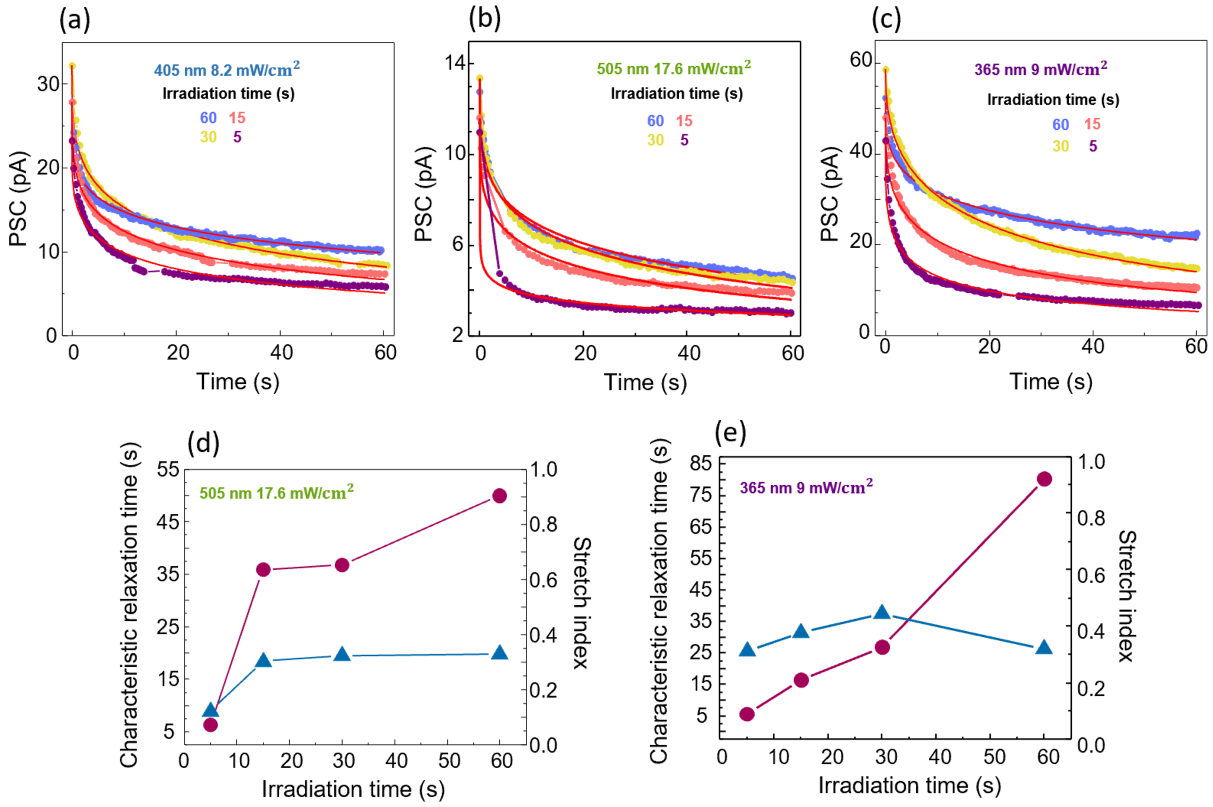

Figure S 12. Kohlrausch stretched exponential PPC fitting on different irradiation times for (a) blue, (b) green and (c) UV light irradiation. Characteristic relaxation time and stretch index for different irradiation times for (d) green light irradiation and (e) UV light irradiation.

In Figure S13, learning and forgetting behavior by pulsing light is shown with 3 cycles. Learning is simulated by applying 30 optical pulses of 150 ms in at 1 Hz, and forgetting is performed in the dark by applying solely the V_{Read} of 0.1 V for 20 s. After the first forgetting process the device has retained 48 % of the learned state. During the second cycle, the memristor needed 4 s to reach the previously learned state and during forgetting it retained 69 % of the state. In cycle 3, only 1 s was required to reach maximum current and the device remembered 78 % of the state by the end of the forgetting process. This indicates harder forgetting and easier relearning by task repetition, once again successfully mimicking the human brain.

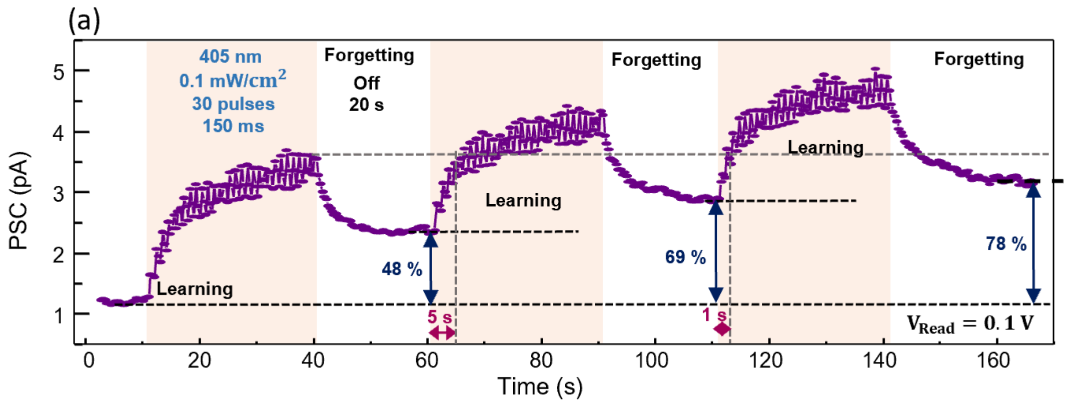

Figure S 13. Learning and Forgetting demonstration by pulsing. Learning is performed by 30 optical pulses of 150 ms, 405 nm wavelength, 0.1 mW/cm^2 of power and 1 Hz of frequency. Forgetting is performed in the dark by applying the V_{Read} of 0.1 V for 20 s.

Table S 1 Comparison on energy consumption of the most energy efficient optoelectronic synaptic devices reported in the recent literature for visible light detection.

Photosensitive layer	Configuration	Size (μm^2)	Light wavelength (nm)	Energy consumption	Ref.
**IGZO/SnO_x/PP**	Planar	400000	400-650	40 pj – 450 nm	1
**Si-NM/$MAPbI_3$**	Planar	12500	532	1 pJ	2
**MoS_2/BTO**	Planar	60	450-650	1.8 pJ – 450 nm	3
**IGZO/$CsPbBr_3$ QDs**	Planar	820 000	350-600	1.35 pJ – 520 nm	4
**$CsPbBr_3$/TIPS**	Planar	16 000	365-500	0.076 pJ -450 nm	5
**DPPDTT/SEBS**	Vertical	N.M.	430	0.12 fJ	6
**Si NM/P_3HT/PbS QDs**	Planar	1000	365-1342	15.8 pJ – 532 nm	7
**PbS QDs+ PMMA/pentacene**	Planar	500 000	365-850	0.55 fJ	8
**ZnAlSnO/SnO**	Planar	200 000	635	0.75 fJ	9
**IGZO:H**	Planar	N.M.	400-1000	2.3 pJ – 500 nm	10
**IGZO:H**	Vertical	4	365-505	0.01 fJ - 405 nm<br>0.009 fJ - 505 nm	This work

(1) Yu, J. J.; Liang, L. Y.; Hu, L. X.; Duan, H. X.; Wu, W. H.; Zhang, H. L.; Gao, J. H.; Zhuge, F.; Chang, T. C.; Cao, H. T. Optoelectronic Neuromorphic Thin-Film Transistors Capable of Selective Attention and with Ultra-Low Power Dissipation. *Nano Energy* **2019**, *62*, 772–780. https://doi.org/10.1016/j.nanoen.2019.06.007.

(2) Yin, L.; Huang, W.; Xiao, R.; Peng, W.; Zhu, Y.; Zhang, Y.; Pi, X.; Yang, D. Optically Stimulated Synaptic Devices Based on the Hybrid Structure of Silicon Nanomembrane and Perovskite. *Nano Lett* **2020**, *20* (5), 3378–3387. https://doi.org/10.1021/acs.nanolett.0c00298.

(3) Du, J.; Xie, D.; Zhang, Q.; Zhong, H.; Meng, F.; Fu, X.; Sun, Q.; Ni, H.; Li, T.; Guo, E. jia; Guo, H.; He, M.; Wang, C.; Gu, L.; Xu, X.; Zhang, G.; Yang, G.; Jin, K.; Ge, C. A Robust Neuromorphic Vision Sensor with Optical Control of Ferroelectric Switching. *Nano Energy* **2021**, *89*. https://doi.org/10.1016/j.nanoen.2021.106439.

(4) Xin, Z.; Tan, Y.; Chen, T.; Iranmanesh, E.; Li, L.; Chang, K. C.; Zhang, S.; Liu, C.; Zhou, H. Visible-Light-Stimulated Synaptic InGaZnO Phototransistors Enabled by Wavelength-Tunable Perovskite Quantum Dots. *Nanoscale Adv* **2021**, *3* (17), 5046–5052. https://doi.org/10.1039/d1na00410g.

(5) Liu, J.; Yang, Z.; Gong, Z.; Shen, Z.; Ye, Y.; Yang, B.; Qiu, Y.; Ye, B.; Xu, L.; Guo, T.; Xu, S. Weak Light-Stimulated Synaptic Hybrid Phototransistors Based on Islandlike Perovskite Films Prepared by Spin Coating. *ACS Appl Mater Interfaces* **2021**, *13* (11), 13362–13371. https://doi.org/10.1021/acsami.0c22604.

(6) Hao, D.; Chen, T.; Guo, P.; Liu, D.; Wang, X.; Huang, H.; Huang, J.; Shan, F.; Yang, Z. Artificial Optoelectronic Synaptic Devices Based on Vertical Organic Field-Effect Transistors with Low Energy Consumption. *Adv Compos Hybrid Mater* **2023**, *6* (4). https://doi.org/10.1007/s42114-023-00712-6.

(7) Wang, Y.; Yin, L.; Huang, S.; Xiao, R.; Zhang, Y.; Li, D.; Pi, X.; Yang, D. Silicon-Nanomembrane-Based Broadband Synaptic Phototransistors for Neuromorphic Vision. *Nano Lett* **2023**, *23* (18), 8460–8467. https://doi.org/10.1021/acs.nanolett.3c01853.

(8) Zhang, J.; Guo, P.; Guo, Z.; Li, L.; Sun, T.; Liu, D.; Tian, L.; Zu, G.; Xiong, L.; Zhang, J.; Huang, J. Retina-Inspired Artificial Synapses with Ultraviolet to Near-Infrared

Broadband Responses for Energy-Efficient Neuromorphic Visual Systems. *Adv Funct Mater* **2023**, *33* (32). https://doi.org/10.1002/adfm.202302885.

(9) Yang, R.; Wang, Y.; Li, S.; Hu, D.; Chen, Q.; Zhuge, F.; Ye, Z.; Pi, X.; Lu, J. All-Optically Controlled Artificial Synapse Based on Full Oxides for Low-Power Visible Neural Network Computing. *Adv Funct Mater* **2024**, *34* (10). https://doi.org/10.1002/adfm.202312444.

(10) Rho, H. Y.; Bala, A.; Sen, A.; Jeong, U.; Shim, J.; Oh, J. on; Ju, Y.; Naqi, M.; Kim, S. Plasma-Engineered Amorphous Metal Oxide Nanostructure-Based Low-Power Highly Responsive Phototransistor Array for Next-Generation Optoelectronics. *ACS Appl Nano Mater* **2023**, *6* (17), 15990–15999. https://doi.org/10.1021/acsanm.3c02973.
